# A Descriptive Morphology of the Ant Genus *Procryptocerus* (Hymenoptera: Formicidae)

**DOI:** 10.1673/031.010.11101

**Published:** 2010-07-15

**Authors:** F. Serna, W. Mackay

**Affiliations:** ^1^ Grupo Sistemática de Insectos Agronomía (SIA). Línea Taxonomía de insectos. Universidad Nacional de Colombia. Facultad de Agronomía. Museo Entomológico Universidad Nacional Agronomía Bogotá (UNAB). Cra. 30 # 45-03. Bogotá, Colombia; ^2^ Department of Biological Sciences. The University of Texas at El Paso. El Paso, TX, USA. 79968

**Keywords:** Hymenoptera, Aculeata, Formicidae, Cephalotini, qualitative characters, external anatomy, taxonomy

## Abstract

Morphology is the most direct approach biologists have to recognize uniqueness of insect species as compared to close relatives. Ants of the genus *Procryptocerus* possess important morphologic characters yet have not been explored for use in a taxonomic revision. The genus is characterized by the protrusion of the clypeus forming a broad nasus and antennal scrobes over the eyes. The toruli are located right posterior to the flanks of the nasus opposite to each other. The vertex is deflexed posteriorly in most species. An in-group comparison of the external morphology is presented focusing on the workers. A general morphology for gynes and males is also presented. Previously mentioned characters as well as new ones are presented, and their character states in different species are clarified. For the metasoma a new system of ant metasomal somite nomenclature is presented that is applicable to Aculeata in general. Finally, a Glossary of morphological terms is offered for the genus (available online). Most of the terminology can be used in other members of the Formicidae and Aculeata.

## Introduction

**Figure 1.**
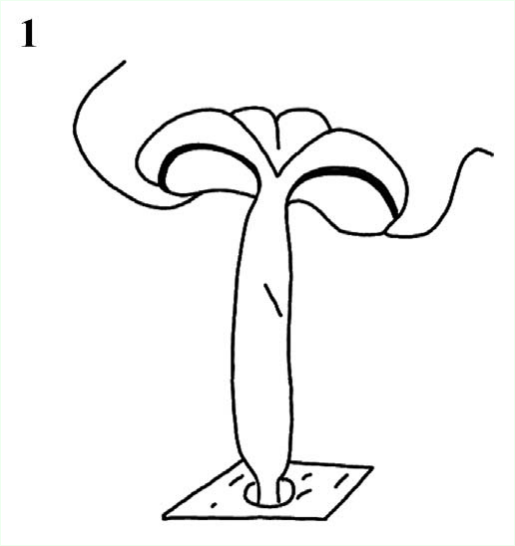
Mushroom-shaped proventricular valves found in the tribe Cephalotini (Redrawn from Emery 1922). High quality figures are available online.

The genera *Procryptocerus* and *Cephalotes* comprise the tribe Cephalotini (de Andrade and Baroni-Urbani 1999. Emery (1922) demonstrated that the tribe possesses the synapomorphic anatomical trait of mushroom-head-shaped proventricular valves (Kempf 1951) (Figure 1). This observation is supported by the studies of de Andrade and Baroni Urbani (1999). *Procryptocerus* was created by Emery (1887) to include species of Neotropical ants that were considered similar to those of the Paleotropical genus *Cataulacus* of the tribe Catalaucini (Kempf 1951). *Procryptocerus* is a lineage composed of about 80 species inhabiting rainforests from the Isthmus of Tehuantepec in Mexico to northern Argentina. Due to their cryptic habits, living inside twigs, these ants are rarely collected (Mackay and Vinson 1989). At present, most species are known from Central America, Colombia and Brazil.

*Procryptocerus* has been the object of two revisionary studies. Kempf (1951) revised the entire genus and Longino and Snelling (2002) the Central American species. Kempf (1951) recognized 28 species, and 8 subspecies, while for Central America Longino and Snelling (2002) recognized 14 species, described four new species, synonymized two species, and elevated two subspecies to species level. Currently, 56 nominal taxa are included in the genus (Bolton et al. 2006).

*Procryptocerus* ants possess notoriously variable morphology. Different characters, such as propodeal spine length, form of the clypeus, type of sculpture, and other such characters vary remarkably, sometimes even within the same species. A diagnostic morphology of the genus is proposed to be used as a template for a revisionary study of the entire group following the recommendations suggested by Bolton (2007): “... [not] make an unwarranted assumption that previous authors have already investigated all the useful characters...”; “Initial dependence on previous publications has a strong tendency to restrict the scope of a new investigation...”; “... develop a personal insight into [the] morphology and variation that is not unduly influenced by what has previously been published.” Knowledge of morphology and anatomy is incomplete for all species. New characters must be discovered, and old characters tested. Morphological descriptions are thus essential components of our understanding of species and their diversification (Wheeler 2008, Bert Hölldobler, Universität Würzburg, personal communication).

This approach is divided into two sections. In the first part, a diagnosis is presented that expands previous morphological diagnoses of the genus provided by Kempf (1951) and Longino and Snelling (2002). Additional observations, current morphological terminology, and figures are part of this new diagnosis. A unification of the terminology is proposed within ants and with other Aculeata regarding the specialized system used to name the metasomal somites (abdominal (Abd) somites II to pygidium) (see Materials and Methods, including definition of *cinctus*).

In the second part, a selected Glossary (available online) is offered containing terminology appropriate for *Procryptocerus*. The provided terminology might also be used for descriptions as well as identification keys of other taxa of ants. Working on the study of morphology and the associated terminology is a constant necessity in order to unify criteria for the basic descriptive work in taxonomy and comparative biology.

## Materials and Methods

Worker, gyne and male specimens of *Procryptocerus scabriusculus* from CWEM (William and Emma Mackay Collection, El Paso, Texas, USA) were drawn at 60X power with a Wild Heerbrugg microscope using a grid and a micrometer. Some structures were cleared in potassium hydroxide (10%) for 36 hours. To analyze the metasomal sclerites, the method in Bolton (1994) was followed. To show differences in sculpturing, structures of different species were also drawn. A diagnosis of males is modified from Kempf (1951). Since few, and in some cases are not available for study males in the genus, this morphological diagnosis emphasizes females and is concentrated in qualitative characters. Terminology used for positions and orientations is explained below. For the study of exoskeletal morphology, the main literature resources were Snodgrass (1935), Bohart and Menke (1976), Gauld and Bolton (1988), Bolton (1994), and Ward and Downie (2005). Sculptural terminology is from Sparks (1941), Harris (1979), Torre-Bueno (1989), Brown (1979) and Hölldobler and Wilson (1990). Vestitural terminology is from Sparks (1941), Hölldobler and Wilson (1990), and Ward (2004). Although *Procryptocerus* ants are mostly black, variation in color is included to help distinguish some forms. Specific terminology is selected from different publications used for descriptions of ants and other Apocritans: Snodgrass (1935), Gauld and Bolton (1988), Nichols (1989), Gordh and Headrick (2000), Hölldobler and Wilson (1990), Mackay (1991, 1993), Bolton (1990, 1994, 2003), Agosti et al. (2000), Longino and Snelling (2002), Mackay and Mackay (2003, 2006), Triplehorn and Johnson (2005), Wilson (2003) and Ward (1999, 2004).

### Terminology

Terminology indicated within parentheses and quotation marks, e.g. (“girdling constriction”) has been avoided. Words within brackets and *italized*, e.g. [*mayri*] are examples of species possessing the specific character state pointed out; accordingly, this bracket use does not indicate that the defined character state is restricted to the examples given. No examples are given when the character state explained is fairly common. Numbers within brackets following the capital letters FS_, [FS_01], [FS_02], [FS_03], etc., indicate species level taxa in the process of being described. When hyphenated sculptural states are used, i.e. “foveoate-costate”, both forms of sculpture are present as intraspecific variation. In the Discussion sculptural terminology is organized as interpreting the form of sculpturing from the smallest to the largest.

**Figure 2.**
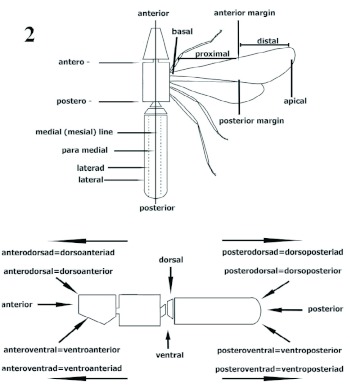
Dorsal (upper), and lateral (lower) representation of a hypothetical insect showing common positions and orientations used to discribe bilateral organisms. High quality figures are available online.

Specific positions such as basal, proximal, distal, apical, apicolateral, apicomesial are used exclusively for appendages such as buccal appendages, antennae, wings, legs, or genital appendages (Figure 2). Indications of positions such as “propodeal base”, “gastral base”, “base of declivity” are avoided since they are referring to structures on the mesion, and not to appendages. Other specific positions (anterior, posterior, dorsal, ventral, lateral, mesial, etc.) are used for body parts; relative positions (with the adverbial ending *ad* meaning toward, such as basad, distad, anteriad, cephalad, posteriad, caudad, laterad, mesiad, anterodorsad, anteroventrad, posterodorsad, posteroventrad, etc.), directionality (mesially, laterally), and extended positions, that involve two or more regions (dorsolaterally, dorsosternally, dorsoventrally, lateroventrally, anteroposteriorly, posteroanteriorly, lateromesially, etc.), are used for both the mesion and the appendages. In relative (*ad*) positions, such as lateroventrad, the prefix “latero” emphasizes that the structure or character state is lateral and the suffix “ventrad” indicates that it is found in the direction of venter. The opposite applies to ventrolaterad or other combinations of prefixes and suffixes indicating relative positions.

In the sense used here, ante means before (anterior to) the referred structure (e.g. antepropodeal refers to a structure anterior to the propodeum), and antero refers to the anterior portion of the actual structure. Anteropropodeum refers to the anterior region on propodeum. Nevertheless, very common literature uses of the prefix “pre” are not changed when, for example, making reference to presclerites as presternite, pretergite, etc.

The use of terminology for shapes is quite useful for describing different structures. A combination of technical and common (not universal) names describing shapes is present in the literature. For instance, terms such as crescentiform, fusiform, disciform, etc. are technical and therefore universal. Terms such as “neck” for a part of the antennae, “cheeks”, “apron”, etc. are not technical, not universal and therefore are avoided.

### **Propodeum** (Figures 9, 10, 15, 37, 38)

The propodeum is the first abdominal tergite fused to the thorax, which together comprise the mesosoma. The propodeum is differentiated into the anteropropodeum and the posteropropodeum. In turn, the anteropropodeum is divided into the dorsopropodeum and the lateropropodeum. The dorsopropodeum is the dorsal area of propodeum, anterior to propodeal spines and containing the anteropropodeal processes laterally. The lateropropodeum is the lateral area (laterotergite) of the anteropropodeum containing the propodeal spiracle. The posteropropodeum is located beneath the propodeal spines; it is the posterior vertical or declivitous area of the propodeum.

### **Metasoma** (Figures 37, 38, Table 1).

In referring to metasomal somites in ants, usually two different systems are superimposed (Bolton 1994). A general system regards homologous abdominal somites (Abd) throughout the Hexapoda. In the Formicidae, as is it in the entire Apocrita, Abd I is part of the second tagma or the mesosoma, which is formed from the thorax plus Abd I tergite. The remaining abdominal somites form the third tagma starting at Abd II (petiole). A second, specialized (functional) system divides the metasomal somites into a petiole, postpetiole and gastral segments (Bolton 1994, 2003). Because different groups of ants contain forms with one- or two-petiolate metasoma, the current specialized system of metasoma nomenclature uses the name “gaster” inconsistently and incongruently with homologous somites in the non-formicid Apocritans. This situation shows that the development of a consistent system of naming specialized metasomata has passed behind the terminology for prosoma or mesosoma. Bolton (1990) introduced the term helcium. A second helcium is characteristic of two-petiolate metasomata; when it is not present, no specialized term is available for Abd III, and hence the term postpetiole is inconsistently used between different castes and subfamilies. Occasionally, authors have had to explain the need of petiole and postpetiole in males without helcial sclerites (see de Andrade and Baroni-Urbani 2003). A simple solution to the inconsistent use of the terminology regarding postpetiole and gastral somites in ants would be to abandon the specialized system of metasoma vocabulary. However, the use of a specialized system has shown interesting advantages in the comparative morphology of ants (see Bolton 2003), and is applied in recent classifications (Bolton 2003, Perrault 2004, Ward 2007). Therefore, improvements to the specialized metasomal terminology are desirable. This work proposes a proposal of reconciliation into a single morphological specialized system for what we believe are homologous metasomata within ants and other Aculeata (Table 1).

**Table 1.**
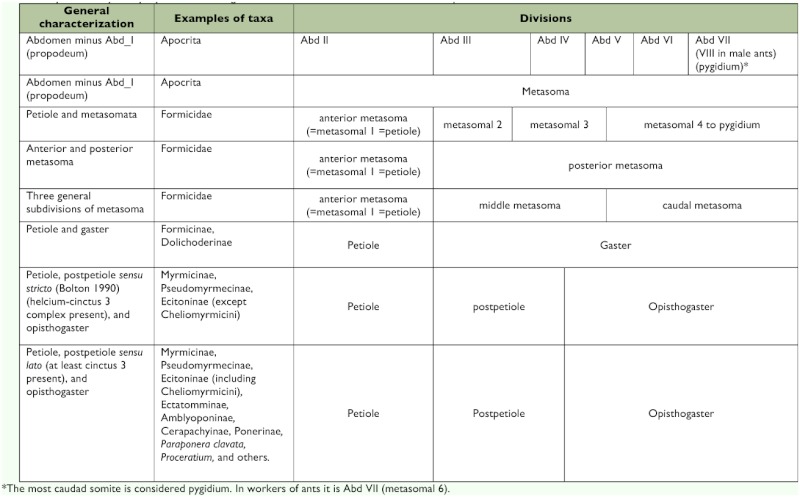
Specialized system proposed for naming metasomata in ants with reference to other Apocritans.

In Apocrita, the metasoma is composed of Abd II to the caudal segment, or periproct (Snodgrass 1935). Literally, “gaster” means stomach (Brown 1979). However, the word “gaster” has widely been used in the literature of Apocrita referring to the external third tagma minus the peduncle (“petiole” in no ants) (Bohart and Stange 1965, Naumann 1991). The gaster constitutes the abdomen without Abd I (propodeum) and the peduncle of Abd II (Naumann 1991).

The Abd II (petiole) is always specialized in ants (Bolton 1994). The petiole is also found in some groups of Tiphiidae, Scoliidae, Vespidae, Mutillidae and other Aculeata, where Abd II is a specialized somite as well. The petiole may be nodiform, squamiform or a much reduced subcylindrical segment (Bolton 1994). Pedunculate, sessile and subssesile petioles in ants are, in general, artifacts of the anteroposterior displacement of the petiolar nodus. In groups with a pedunculate petiole (e. g. *Pheidole* or *Solenopsis*), the node is posterior. In sessile petioles (such as in *Procryptocerus*), the tergum is augmented anteriorly (nodal) usually forming an anterior nodal truncation.

The metasoma comprises two general subdivisions: the anterior metasoma constituting the petiole (Abd II), and the posterior metasoma composed of metasomal (mtm) 2 (Abd III) to pygidium. In the posterior metasoma, Abd III–IV (mtm 4–5) constitutes the middle metasoma, Abd V to pygidium constitutes the caudal metasoma. The petiole (mtm 1) contains the cinctus 1 (cinctus: constriction between pre and postsclerites, see below), the mtm 2 contains the cinctus 2, and the mtm 3 may or may not contain the cinctus 3. The petiole (Abd II) and mtm 2 (Abd III) are joined by the first helcium (Bolton 1990) in all ants. In the middle metasoma, the mtm 2 and 3 (Abd III– IV) may or may not be joined by a second helcium. The mtm 2 is usually considered the postpetiole when separated from mtm 3 by the helcium-cintus 3 complex. This state is seen in females of Myrmicinae, Pseudomyrmecinae, and others. In other considerations of a postpetiole, the mtm 2 is separated from mtm 3 by the cinctus 3 only (e.g. see de Andrade and Baroni-Urbani 2003 for *Proceratium*). This state is known to occur in males of Myrmicinae, Pseudomyrmecinae, and in both sexes of Ecitoninae (including Cheliomyrmicini), Ectatomminae, Amblyoponinae, Cerapachyinae, Ponerinae, *Paraponera clavata, Proceratium*, and others.

For the posterior metasoma, we propose the use of the name gaster when a postpetiole is not present (e.g. Formicinae, Dolichoderinae), and the use of postpetiole and opisthogaster when at least a cinctus 3 is present. The posterior metasoma is a gaster when its metasomata 3 to pygidium (Abd IV to pygidium) are combined as a unit (e.g. Formicinae, Dolichoderinae); i.e. the gaster constitutes the metasoma minus the petiole when a postpetiole is absent. The opisthogaster constitutes the combined caudal metasomata 4 to pygidium (Abd V to pygidium); i.e. the opisthogaster is the posterior metasoma minus postpetiole. Even in few cases, when it is not clear to associate gaster and opisthogaster to general subfamilies, tribes or genera, the two terms would stay associated to the already explained metasomata.

In summary, we propose a consistent specialized (functional) and morphological system for naming homologous metasomata in ants. When the cinctus 3 is absent in the metasomite 3 (Abd IV), we propose using gaster for the combined metasomata 3 to pygidium. When a postpetiole is considered, due to the presence of at least the cinctus 3, the posterior metasoma is divided into postpetiole and opisthogaster.

### **Cinctus 1, 2, 3** (pl. **cincti**) (Figures 3, 9, 16).

Cincti are the anteriad sulci, often in the shape of a belt or girdling constriction, located on metasomal 1 (Abd II = petiole), metasomal 2 (Abd III = postpetiole) and metasomal 3 (Abd IV) somites. The sulci separate the pre and post sclerites. Cinctus 1 is usually a dorsal, very slender sulcus concealed between the posteropropodeal lobes, anterior to the peduncle and sternopetiolar process in petioles possessing these structures. In some groups (e.g. *Pogonomyrmex*) there is no apparent cinctus 1. In such cases, cinctus 1 is interpreted as the anteriormost portion of the peduncle located between the posteropropodeal lobes. In general, in sessile metasomata (e.g. *Procryptocerus*) cinctus 1 is located directly anterior to the spiracle. The cinctus 2 is located in metasomal 2, anterior to the ventropostpetiolar (“subpostpetiolar”) process in metasomal 2 that possesses such process. The cinctus 3 (the “girdling constriction”- Bolton 1994) is located between the pre and postsclerites of the metasomal 3, posterior to the second helcium (*sensu* Bolton 1990), in two-petiolate ants. When the cinctus 3 is present in some of the *sensu stricto* single petiolate ants, it is a very fine sulcus (e.g. different poneroids groups), or almost absent (e.g. *Odontomachus*). Few genera such as *Leptanilliodes* possess more than three cincti.

## Results

### Workers

#### Diagnosis

**Figure 3.**
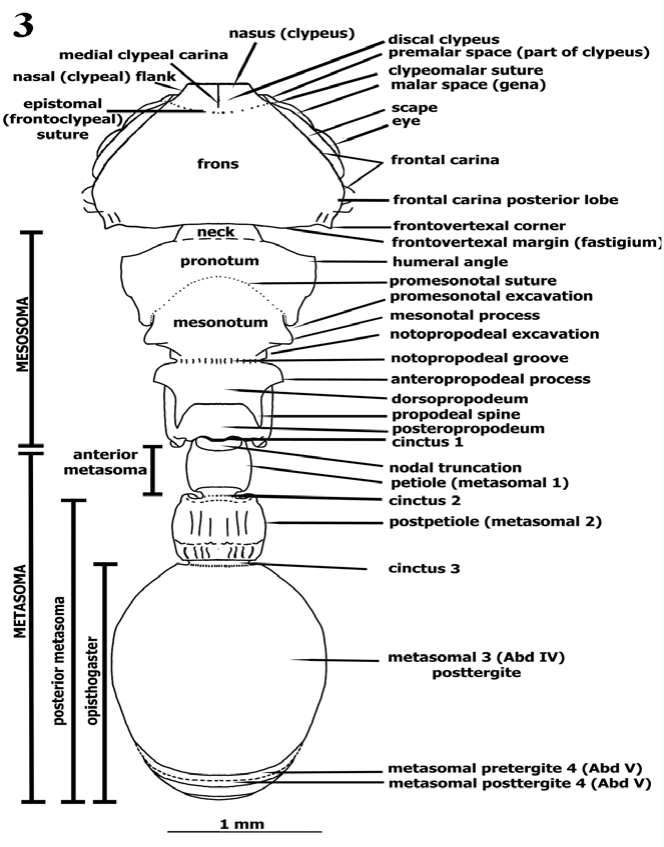
*Procryptocerus scabriusculus*. Dorsal view. High quality figures are available online.

**Figure 4.**
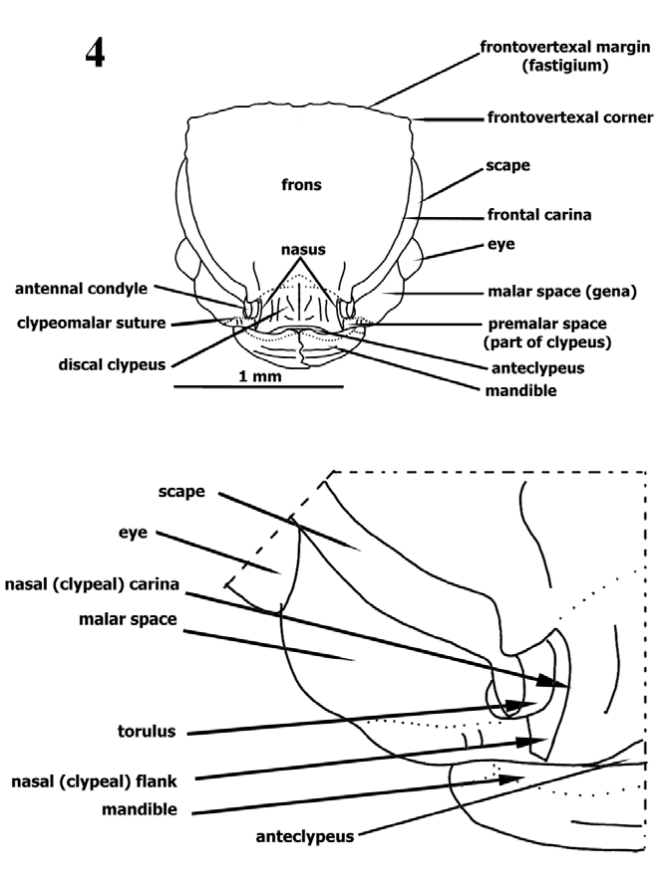
*Procryptocerus scabriusculus*. Worker. Head, dorsoanterior (frontal) view. Above: face. Lower: half side of face zoomed (part) to show morphological details. High quality figures are available online.

Frontal carinae posteriorly divergent; malar space not covered dorsally by frontal carinae; clypeus protruded developed into a broad nasus lying between toruli; antennal scrobes impressed laterally above eyes, limited by frontal carinae dorsally (Figures 3, 4), extending from nasus to vertex; vertex deflexed (truncate) posteriorly in most species; toruli located right behind nasal flanks opposite to each other; Abd V postergite visible from above. Adult coloration mostly black, body variously sculptured; workers monomorphic, ranging from 3.5–8.5 mm (Kempf 1951), gynes from 3.7 mm [*schmitti*] to 9.5 [*mayri* part].

#### **Head** (Figures 3, 4, 5, 8, 9)

**Figures 5–8.**
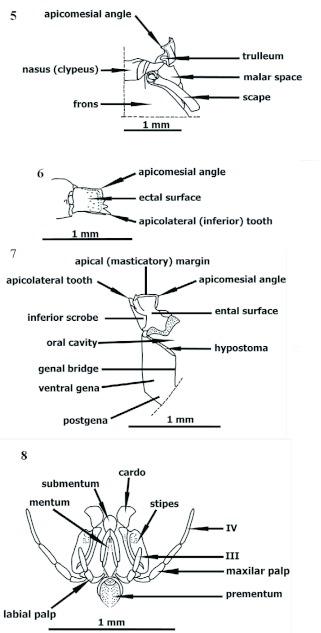
*Procryptocerus scabriusculus*. Worker. Buccal pieces. 5–7 right mandible: 5: dorsal, 6: lateral, and 7: mesial views; 8: posterior view of maxilolabial complex. High quality figures are available online.

**Figure 9.**
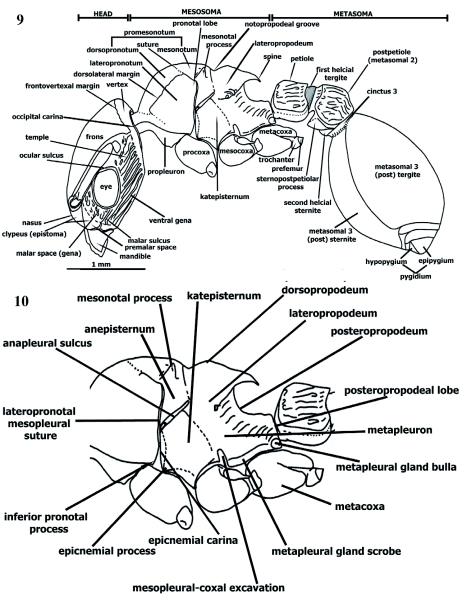
*Procryptocerus scabriusculus* Worker. Lateral (profile) view. **Figure 10.** *Procryptocerus scabriusculus*. Worker. Mesosoma profile zoomed to show details. High quality figures are available online.

Prognathous, usually in anteroventrad position in preserved specimens (Figure 9); prognathism formed by combination of hypostomal, genal and postgenal bridges. In full frons (“full face”) view, subtrapezoidal, broadened posteriorly (Figure 4), circular [*nalini*] (Figure 23), subcircular [*regularis, pictipes*] or subquadrate [*belti* (Figure 20), FS_02]; in lateral view, more often elliptic; mandibles, anteclypeal carina and clypeus anteriorly positioned; frontoclypeus (“face”) anterodorsally extended, comprising epistoma (anteclypeus, plus clypeus) and frons; frons dorsal; vertex posterior, opposite to nasus, often orthogonal to frons (deflexed); in dorsal (full frons) view, frontoclypeal (epistomal) suture and frontovertexal margin located in nearly same plane; malar space (Gauld and Bolton 1988) extended dorsolaterally, laterad to antennal scrobe and anteriad to eye (Figures 3, 4, 9); hypostomal bridge and genal bridge ventral (Figure 7), opposite to frons, occiput and occipital foramen posteroventrad, postgenal bridge ventroposteriad, posterior to genal bridge, with the two areas meeting in a curve or an angle behind eye; anteclypeal carina (Figure 4) indistinct, emarginate, or bilobate; clypeus differentiated into discal (central) and truncate strikingly orthogonal lateral nasal flanks toward toruli (Figures 3, 4, 9) [*eladio, pictipes, goeldii, marginatus*], or trapezoidal, not orthogonally flanked, being wider on lower lateral area toward malar space and forming invaginated antennal fovea (Figure 24) [*batesi*]; frontoclypeal (epistomal) suture obsolete between toruli, turning down laterad between nasal flank (located anteriorly) and torulus (located posteriorly); epistomal suture lateral between clypeus and torulus, not grooved [*paleatus*], faintly grooved [*mayri*], or variably grooved and forming clypeo-torular sulcus [*carbonarius*, some *rudis*] (Figures 9, 24), continuing downward to pleurostoma, anteriad to malar space, sometimes indistinct and forming vanishing clypeomalar (clypeogenal) suture (Figures 3, 4) separating narrow premalar space from malar space; anterior tentorial pit located into clypeomalar suture, anterior to lateral fovea (Gordh and Headrick 2000); nasal flanks anterior to toruli; discal clypeus protruded into broad, nasus (Figures 3, 4, 9) in approaching frons; nasus describing in profile short anterodorsal curve at toruli level; lateral fovea beneath torulus (not visible in dorsal view) and posterior to clypeomalar suture, marking antennal scrobe at most anterior point and receiving lamella of scape shaft base; facial fovea (malar depression), when present [*mayri, batesi, carbonarius, rudis*] (Figure 24) encircling part of clypeogenal suture, and often part of antennal fovea (Torre-Bueno 1989); malar tumulus (Figure 24) anteriad to eye and limiting facial fovea laterally [*batesi, rudis*]; frons anteriorly delimited by faint frontoclypeal (epistomal) suture or faint frontal triangle (Figure 4), laterally by frontal carinae, posteriorly by frontovertexal margin; frontal carinae diverging from clypeus to vertex, straight, uni or bilobate [*mayri, batesi*] (dorsal view) into frontal lobe and frontal carina posterior lobe(postfrons lobe), posterior lobe deflexed posteriad in some species forming an angulate scrobe [*rudis*] (Figure 24); frontal lobe indistinct (not flanged) (Figures 3, 4, 9, 17–19, 23, 29) [*scabriusculus, hylaeus*, brazilian species] or distinct [*rudis, mayri, batesi*] (Figures 20–22, 24); antennal scrobe lateral, under frontal carina, not visible in full frons view, formed of long, deep, wide lateromesial invagination (groove), extended anteroposteriorly, starting at lateral fovea, passing over eye, and terminating in notch on vertex at frontovertexal corner level (Figures 9, 24), dorsally delimited by frontal carina and ventrally by dorsal ocular suture or anteocular costula; eye lateral, infra antennal scrobe (head in profile), slightly posteriad, or intermediate between torulus and vertexal margin; eye globular [*convergens, rudis*], flat [FS_03], rounded not-protruded [*marginatus*], ellipsoid [*scabriusculus, kempfi*], dorsally depressed [*adlerzi, sampaioi*]; vertex deflexed, often delimited anteriorly from frons by transverse frontovertexal margin, posteriorly by occipital carina, and laterally by posterior notches of antennal scrobes [*batesi, rudis, mayri, virgatus*]; frontovertexal margin distinct throughout forming fastigium (Figures 3, 4, 9, 14, 15, 16–19, 25, 27, 29) [*impressus, paleatus, regularis, adlerzi, scabriusculus*], indistinct throughout [*coriarius, schmitti*] (Figure 23), medially indistinct and laterally distinct [*mayri, clathratus*] (Figures 20–22), or frontovertexal margin outline (frontal or posterior view) straight [*eladio, kempfi, subpilosus*], slightly medially notched [*scabriusculus* in part, *convergens*] (Figures 4, 19), crenate [*clathratus, impressus, marginatus, spiniperdus*] (Figure 18), crenulate [*paleatus*], convex [*coriarius, nalini*] (Figure 23), slightly convex-crenulate [*paleatus*], biconvex [*adlerzi*] or flanged-biconvex [*adlerzi*] (Figure 27); frontovertexal corner with (Figures 3, 4, 9, 16, 17, 20, 22, 27) or without [*coriarius,nalini*] (Figure 18) angulate processes; vertex concave [*clathratus, marginatus, paleatus, impressus, spiniperdus*], flat [*scabriusculus, lepidus, balzani*] or slightly convex [*coriarius*]; hypostomal tooth lobose. Mandible (Figures 5–7) subrectangular, possessing scrobe ventrally and trulleum dorsally, massive, medially turned; inferior margin (lateral view, mandible closed) ventrad [generally species at low elevations], anteroventrad [most Andean species over 600 m]; apical (“dentical”) margin medially directed, possesing major distinct, infra, apicolateral (“apical”) tooth; preapicolateral tooth (tooth number 2) half size of apicolateral; tooth number 3 [obsolete in *pictipes, mayri*] half size of number 2 [FS_01]; tooth 4 obsolete or absent; supra, apicomesial (“basal”) angle acute. Maxillarlabial palp formulae 4–3 [FS_01, *carbonarius*] (Figure 8), 6–3 (Kempf 1951); palpifer almost half size of first palpomere; first and second palpomeres same size, apical (fourth) longest. Antenna 11-segmented; scape comprises short, basal radicle and long, distad shaft; radicle divided into condylar bulb (Bolton 1994) (Figures 4, 14) (inserted into torulus) and condylar constriction (“neck”), which functionally fits into posterior notch of torulus during anteroposterior movement of scape; condylar bulb anteriorly visible [*attenuatus, victoris, seabrai*, FS_03] or relatively concealed by torulus [*carbonarius*]; scape stalk truncate basally [*attenuatus*], often proximally terete (narrow), wide [*attenuatus, nalini*], or slightly tapered [*scabriusculus, convergens*], basally uni or bilamellate (broad, thin, flanged carina) overlapping condylar constriction [*rudis*], or ecarinate [*schmitti*]; scape lateral axis (lateral stalk) not completely covered by frontal carina when accommodated into antennal scrobe; scape shorter than scrobe length, terminating between eye and vertex; funiculus 10-segmented, second funicular segment shorter than first or third, three distal segments compose club, apical segment subconical.

#### **Mesosoma** (Figures 3, 9–11, 26–30)

**Figures 11–13.**
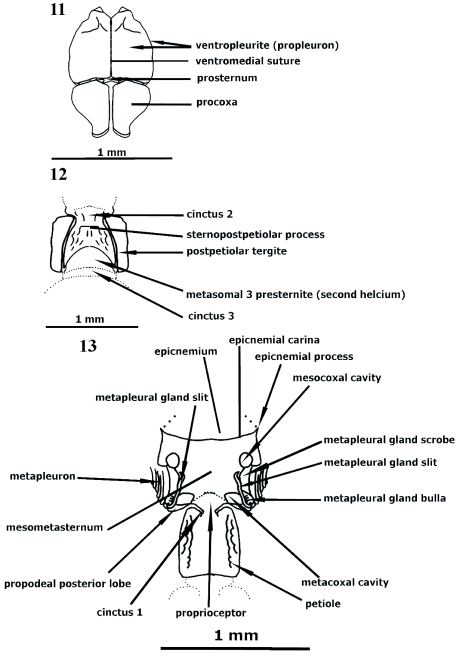
Worker. Mesosoma and waist region. Ventral view. High quality figures are available online.

Subcubic, trunk-shaped, dorsally convex or flat. Tergal, pleural (meso and meta) and propodeal sclerites fused into notosternal-propodeal capsule. Pleura subparallel or mesially inflected between meso and metapleura. Pronotum hood-shaped, covering more than 1/3 of dorsal and lateral areas of mesosoma, forming dorsopronotum and lateropronotum (= pronotal side panel - Gauld and Bolton 1988) (Figure 9); dorsal profile from markedly convex [*gibbossus*] to horizontal (dorsally flat) [*paleatus, impressus*]; dorsopronotum and lateropronotum divided by pronotum dorsolateral margin (Figure 9); lateropronotum subtriangular with vertex at level of procoxal base, sometimes forming inferior lamella (inferior pronotal process Figure 10) flanking procoxa basally [Andean species], both structures (inferior pronotal process and procoxa base) probably forming stridilum; lateropronotum overlapping propleuron in most species (Figure 9) and forming pronotal lobe posteriorly (Figure 9), lateropronotum mesially inflected conforming humeral angle anteriad (Figure 3) and humerus (elongate, narrow anterior area connecting with neck dorsally); humeral inflection forming lateral carina limiting humerus and lateropronotum (wide lateral panel); lateropronotum flat discally (Figure 9) or slightly inflected (Figures 29, 30) receiving disciform profemur [*impressus*], both structures probably forming stridulating organ; ventro-propleurites separated by ventro medial suture (Figure 11), sometimes apparently fused; ventropropleurite and humerus forming protruded or flat area coupling postgenal bridge when head deflexed downwards; prosternum between procoxae, posteriad to ventropropleurites; mesonotum flat [*pictipes*] or convex [*sampaioi*], laterally fused to anepisternum (Figures 9, 10), anteriorly delimited by vestigial promesonotal suture (Figures 3, 9, 26, 27), extending posteriad until meeting *notopropodeal fusion* (see Discussion), usually possessing lobose or spiniform lateral process (mesonotal process) (Figures 3, 9, 27); promesonotum possessing lateral excavations between pronotal lobe and mesonotal process; lateropronotum and mesopleuron separated by open, narrow, nearly straight or sinuate lateropronotal-mesopleural suture continuing dorsally, forming promesonotal excavation between pronotal lobe and mesonotal process, connected to promesonotal suture (Figure 3); anapleural sulcus (“anterior oblique sulcus”) (Figure 19) down promesonotal excavation, dividing smaller anepisternum supra and larger katepisternum infra (Figures 9, 10); katepisternum extended lateroventrally, ventrally forming epicnemium (Gauld and Bolton 1988) separating pro and mesocoxae; epicnemial carina (“omalus” sensu Bohart and Menke 1976) (Figures 10, 12) and epicnemium forming shallow-concave surface receiving procoxa posterior face; epicnemial carina projected anteriorly into distinct laminate, variable shaped, subcircular [most Andean species], truncate, square [*adlerzi, regularis*], or falcate [*victoris*] epicnemial process (Figures 10, 12, 30) flanking procoxae externally, both structures probably forming stridulating organ; notopropodeal fusion often marked by groove and lateral excavations [*spiniperdus, eladio, adlerzi*] (Figure 3) or lateral excavations only [*sampaioi*]; mesopleuron usually inflected (mesosoma constricted) receiving femora downward notopropodeal excavation; metapleural gland scrobe (Figures 10, 12) superior to metacoxa, extending posteroanteriorly from metapleural gland bulla (Figures 10, 12) to mesopleural-coxal excavation (mesopleural coxal process Snodgrass 1935- inflected into excavation) (Figure 10); metapleural gland scrobe canalicular, channel delimited superiorly and inferiorly by two longitudinal carinae, inferior carinula flanking metapleural gland slit ventrally; metapleural gland extending dorsoposteriorly, turning downward ventroanteriorly forming metapleural gland slit (Figure 12); slit very narrow, bicarinulate, running ventrally posteroanteriorly from metapleural bulla to mesopleural-coxal excavation; propodeum (first abdominal (Abd I) tergite) divided into anteropropodeum and posteropropodeum, first subdivided into dorsal (dorsopropodeum) and lateral (lateropropodeum) areas (Figures 9, 10); lateropropodeum including spiracle, posteropropodeum (declivitous face) (Figures 9, 10) under propodeal spines terminating at posteropropodeal lobes (propodeal lobes) (Figure 12); dorsopropodeum usually horizontal in profile [FS_01, *scabriusculus*] to convex [*hylaeus, sampaioi*], same plane as mesonotum [*pictipes, lenkoi, sampaioi*] or lower than mesonotum [*clathratus*], expanded anterolaterally into somewhat anteropropodeal process (Figure 3); propodeal spines (Figure 3) horizontal and parallel [*kempfi*], divergent and upturned [*rudis*], parallel upturned [*clathratus*], parallel upturned-curvate [*eladio*], parallel down-turned [*scabriusculus* part] (Figure 9), or different sizes within same population [*scabriusculus* part]; propodeal spiracle tubulose, downward on lateropropodeum between anteropropodeal process and propodeal spine base, commonly directed posteriorly, often accommodated into lateropropodeal excavation, internal margin generally fused to excavation, lateral margin usually free; posteropropodeum sometimes forming continuously concave descending declivity until reaching posteropropodeal lobes, somewhat vertical or slightly diagonal supra, shorter infra (between posteropropodeal lobes), supra and infra areas rarely same length [*scabriusculus* part] (Figure 9); posteropropodeal lobe posterior to bulla (Figure 12); metacoxal cavity ental (Figure 12); legs similar to male's (Figure 47), procoxa trunk-like augmented basally, twice size of meso or metacoxa; profemur tectiform (roof-like), securiform (triangular) in cross section (clear vertices on inferior side), equilateral, or ventral side narrower than anterior and posterior sides [*belti, eladio*], fusiform (spindle-shaped) [*mayri, batesi*], or compressed disciform (disc-shape) [*impressus*] with dorsal margin carinate (keeled) [*impressus, paleatus*] and concave anteromesially (entad) and proximally [*impressus, paleatus*], or convex; slightly convex posterolaterally; meso and metafemora commonly tectiform, ventral side weakly concave, separated from trochanter by small, dorsad, cuneiform prefemur (Kukalová-Peck 1991) (“trochantellus”) (Figures 9, 47), (male profemur elongate fusiform (Figure 47)); tibia subcylindric, possessing four poorly defined panels, anteriad and posteriad wider than ventromesiad (flexor) and dorsolaterad (extensor); foretibia possessing strigil (pectinate curvate spur) ventrodistally forming antenna cleaner with curvate and pectinate probasitarsus; meso and metabasitarsi cylindrical and longer; postarsus (“pretarsus”) formed by bilobed padded arolium and bifurcate curvate claw.

#### **Metasoma** (Figures 3, 9, 12, 13, 15, 16, 26–34, and Table 1)

(Justification of terminology used here for third tagma in Metasoma can be found in Materials and Methods and the Glossary [available online]). Petiole (metasomal 1 = Abd II), first specialized metasomite articulated to propodeum by manubrium (Perrault 2004), composed of tergite and sternal presclerite (Figure 12), forming syntergosternite (tergite and sternite fused), sessile, constricted anteriad into cinctus 1 (cinctus: constriction between pre and postsclerites, see under Metasoma in Materials and Methods) (Figure 12), nodiform, narrower than distance between propodeal spine bases, subcylindrical [*kempfi*], slightly wider anteriad, or barrel-shape [*eladio, batesi*], usually without dorsal or lateral excrescences or projections; node anterior face reduced [*nalini*] or more commonly truncate forming nodal truncation (Figures 3, 32) opposite to and functionally received by posteropropodeum, often delimited by nodal dorsolateral margen; nodal truncation convex, straight, curvate supraposteriad [*adlerzi*], concave [*coriarus, sampaioi*], or absent [*nalini*], petiolar summit anteriad (Figures 3, 29, 31), midway [*hylaeus*] or posteriad [*seabrai*]; sternopetiolar (“subpetiolar”) process between cinctus 1 and node (Figures 29, 31), and usually laminar-lobose [*scabriusculus, rudis, mayri*] or obsolete, petiole posterior foramen margin (lateral view) sinuate (Figures 9, 10) or vertically set off, spiracle anteroventrad. Postpetiole, second specialized metasomite (metasomal 2) (Abd III) (Figure 3), wider than petiole, anterior foramen vertically set off, formed of first helcium (Bolton 1990), adjusted into posterior petiolar foramen, posttergite largest sclerite of postpetiole generally subfungiform (Figures 3, 9, 32, 34), posteriorly augmented forming postnodus (Figure 3), usually with anterolateral lobes [*belti*] posterolaterad to cinctus 2; postnodus usually composed of dorsal and posterodorsad faces [*scabriusculus, clathratus, rudis*], continuously convex posteriad (the two faces not differentiated by postnodus) (Figure 31) [*convergens*], or dorsally flat, narrowing into postnodus and somewhat vertically set off forming lamella posteriorly [*mayri, batesi*]; tergite and sternite separated by dorsosternal sutures, fused (Bolton 2003); poststernite crescentiform, emarginate posteriorly, leaving helcial metasomal 3 (Abd IV) presternite visible (Figures 9, 13, 16), projected anteromedially into sternopostpetiolar (“subpostpetiolar”) process (Figure 9); sternopostpetiolar process forming with cinctus 2 (Figure 13) transversal (trans-sternal) cavity ventrally, where petiolar sternite posterior margin couples; ventropostpetiolar process somewhat conic, transversally truncate (Figure 13), blunt [*spiniperdus*], unilobate [*mayri*] or bilobate apically; caudal postpetiolar foramen posteroventrad giving posteroventrad position (lateral view) to opisthogaster (metasomal 3 [Abd IV] to pygidium, see Metasoma in Materials and Methods) (Figure 3); postpetiolar spiracle anteriad, slightly ventrad. Metasomal 3 (Abd IV) (Figure 3), largest metasomite, first opisthogastral somite, third specialized (possessing second helcium and third cinctus) metasomite occupying nearly 2/3 of metasoma; elliptical or ovate; presclerites forming second helcium (Bolton 1990) (Figure 3); stridulatory organ (Wheeler 1984) formed between metasomal 2 and second helcium; helcial sclerites and postsclerites separated by cinctus 3 (“girdling constriction”) (Figures 3, 9); postergite and poststernite largest metasomal sclerites comprising approximately 3/4 of opisthogastral region; posttergite convex (Figure 9) or slightly depressed [*rudis*]; spiracle anteriad, subdorsad. Metasomal 4 to pygidium somites non-specialized, pre- and posttergites differentiated by pronounced carina (Longino and Snelling 2002); pygidium (Abd VII) divided into epipygium (tergite) and hypopygium (sternite) (Figures 3, 9).

#### **Gynes** (Figures 14–16)

**Figure 14.**
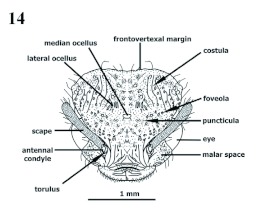
Gyne. *P. scabriusculus*. Head frontal view. High quality figures are available online.

**Figure 15.**
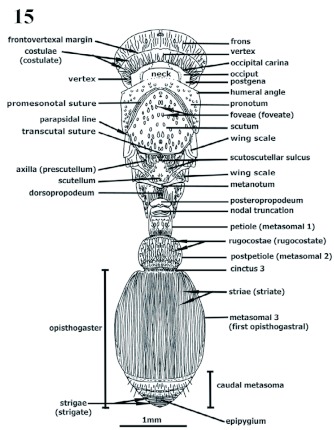
Gyne. *P. scabriusculus*. Dorsal view. High quality figures are available online.

**Figure 16.**
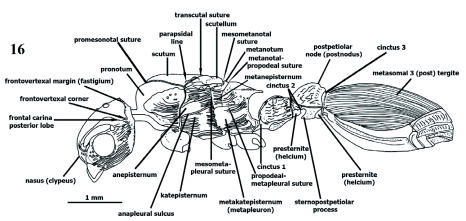
Gyne. *P. scabriusculus*. Lateral (profile) view (habitus). High quality figures are available online.

Although similar to workers, gyne are larger with thoracic sclerites (Figures 15–16) corresponding to alates in Apocritans. Ocelli posteriad within frons (Figure 14).

Gyne variations on mesosoma (Figures 16, 17). Mesonotum divided into anterior scutum and posterior scutellum by curvate, scutoscutellar, or prescutellar groove; scutum divided by transcutal suture into greater anterior sclerite, and posterior prescutellar region, dorsomesial between axillae; parapsidal lines extended posteroanteriorly from transcutal suture to discal scutum, slightly diverging anteriorly; axillae laterally longer and wider, forming prescutellum and embracing scutellum anteriorly; axillular scrobe (“fossa”), where wings rest, lateral, under axilla, formed of lateromedially impressed axillar groove, running from wing axillar sclerites to scutellum; mesoanepisternum and metanepisternum separated by lateral mesometathoracic (mesometapleural) suture; metanepisternum and metakatepisternum separated by short metanapleural sulcus. Wings similar to those of the male.

#### **Sculpture** (Figures 15, 17–34)

Sclerites usually exhibit combination of two or three sorts of sculpturing. Sculpture in *Procryptocerus* divided into microsculpture (background sculpture), and macrosculpture (regular [circular] or irregular depressions, or longitudinal and transverse elevations). Microsculpture: micropunctate, microreticulate, microimbricate or microstrigulate. Macrosculpture: impressed holes without costae, ridges or carinae (foveate, foveolate, punctate), at level of surface (shallow) (striolate, imbricate, areolate, dotted, puncticulate), raised (costate, carinate, carinulate, vermiculate, striate, sulcate, strigate), or their combinations (scrobiculate, porcate, alveolate, rimose). Often, when integument smooth and polished (shiny = glossy), dorsum, especially on metasomal 3 sclerites, micropunctulate and bears combination of other micro sculpture.

Surfaces normally without sculpturing: torulus, hypostoma, funicular segments, and postocciput. Surfaces regularly micropunctate, and without macrosculpture, neck, prosternum, mesonotal lobes, ventral metepisternum, propodeal spines, posteropropodeum, sternal petiole and postpetiole, and metasomal 3 in Andean species over 600 m of elevation. Surfaces microreticulate or microimbricate, without macrosculpture: scape (almost always microimbricate), femora (microimbricate or microstriolate). Elevated ridges (costae and carinae) often microsculptured (micropunctate or microimbricate) on background. Striations and sulcations more common in Brazilian species. Circular impressed sculptures and combination with costae, striations and sulcations more common in Andean and Mesoamerican species.

Frons clathrate in Andean species [*mayri, batesi*] and Brazilian *clathratus* (Figures 21, 22) or foveolate (*schmitti, coriarius,nalini*); metasomal 3 punctate [*belti, impressus* (Figure 33)], glossy [*eladio, belti, mayri, attenuatus, convexus, carbonarius-*postenad], or finelly striate (Figures 15, 32) [some Andean, Mesoamerican, and northern South American species] [*scabriusculus, tortuguero, marginatus, spiniperdus, ferreri*]. Alveolate sculpture of Andean and Mesoamerican species on frons posteriad, pronotum anteriad, tergal petiole [*eladio*, FS_11] and postpetiole (Figure 31); mesosoma, petiole and postpetiole porcate [*batesi, mayri*], tergal postpetiole rugocostate [Brazilian species] [*regularis, sampaioi, convergens, schmalzi*], femora costulate or costate in Central American [*paleatus, impressus*] and Brazilian [*schmalzi*] species.

Clathrate sculpture on frons and promesonotum [*carbonarius, rudis, batesi, mayri, clathratus*] (Figures 21, 22); costate or costulate sculpturing often on mandibles, clypeus (nasus), nasal flanks, frons (Figures 14, 15, 18), malar space, temple, vertex (Figure 15), gena, promesonotum, discal lateropronotum, mesopleuron, propodeum, coxae, femora, tibiae, and metasomal tergites 1, 2, 3; rimae (ondulate striae or costae) more common on frons [*sampaioi, victoris, convergens*] and mesonotum [*victoris*], when metasomal tergite 3 punctulate (densely punctate), some species have farinose texture [*impressus, belti, subpilosus*]; scrobiculae often bordering areas as vertex [*mayri, schmitti, clathratus*], temple [*eladio*], lateropronotum posteriad [*scabriusculus*], mesepisternum anteriad [*scabriusculus*], notopropodeal fusion when grooved [*schmitti, coriarius*], petiole and postpetiole posteriad, cincti 2 and 3 (Figures 15, 32).

Mandible often longitudinally costulate (Figure 4); anteclypeal region often strigate, discal clypeus ecarinate, variably longitudinally costate, or with medial carina or costa (Figures 3, 4), nasal flank ecarinate or costulate; clypeal carina often extending back and continuing mesiad, parallel and very close to frontal carina; frontal carina describing more or less straight line [*eladio*], curvate [*scabriusculus*], *convergens, regularis, subpilosus, coriarius*] (Figures 3, 4, 14), sinuate [*belti*] (Figure 20) or sinuate-bilobate [*mayri, batesi*]; frons foveolate (or foveate) [*nalini, eladio, pictipes*-antenad] (Figure 23), foveate-costulate [*scabriusculus*] (Figure 14), striate [*adlerzi*] (Figure 17), costulate [virgatus], costate [*regularis*], reticulate or areolate [*belti, hirsutus, convexus, pictipes*] (Figure 20), reticulate [*pictipes*-anteriad], areolate [*pictipes*-posteriad], clathrate and areolate [*batesi*] (Figure 21), infra lateropronotum porcate or costate [most species] (Figure 29); mesopleuron porcate or costulate (Figure 29), and foveolate; propodeal spines microsculptured or ecarinate and glossy, posteropropodeum supra strigate [*hylaeus, mayri*], ecarinate and shiny [*mayri*], or striate (or longitudinally costate) [*montanus, striatus*]; meso and metapleura usually costulate (Figure 29); nodal truncation ecarinate and glossy [*belti*] or strigate [*scabriusculus*]; tergal petiole and postpetiole areolate [*coriarius*] (Figure 31); sternal petiole and postpetiole ecarinate [most species]; metasomal 3 tergite striate-costate (Figure 15), costulate, or costate [several species in the whole range of the geographical distribution] (Figures 15, 16, 32); metasomals 4–6 pretergites smooth, postergites strigulate or microtuberculate; epipygium punctulate; metasomal 3 sternite ecarinate [most species], striate (or costulate) [*ferreri*] (Figure 34).

#### **Vestiture** (Figures 22, 26, 30, 31, 35)

Two kinds of vestiture are present in *Procryptocerus*. Pilosity (Figures 26, 30, 31) refers to long, erect, sub erect, sub decumbent, decumbent (Figure 35), or appressed (not drawn) hairs, and pubescence (not drawn) refers to exceptionally short, fine hairs forming second layer beneath pilosity (Hölldobler and Wilson 1990).

*Procryptocerus* bear both short and long, flexous (flagellate) pilosity (Figure 26), or short and long, stiff or subspatulate pilosity (Figures 22, 30, 31); longest pilosity on tergal petiole and postpetiole [*mayri, impressus*,]; often medial dorsal line of meso and metasoma denudate; lateral hairs of anteclypeus pecten (Figure 22) commonly convergent; frons more common with stiff, scattered pilosity; frons posteriad and frontovertexal margin usually with two transverse lines of uniformly separated stiff hairs slightly directed anteriorly; eyes denudate; malar space often possessing few scattered hairs; shorter flexous pilosity or pubescence on postgenal bridge; mandible with erect short hairs, ventrally and distally with flexous pilosity; scape usually with short, stiff, uniformly distributed, sparse hairs (Figure 22) promesonotum and dorsopropodeum with long stiff or flexous hairs; propleuron denudate or pubescent; meso and metapleura usually denudate; coxae both pubescent and with flexous hairs on ventral, dorsal and posterior faces, usually medially pubescent combined with some flexous, sparse hairs; dorsum of femora and tibiae usually with decumbent and subdecumbent, stiff hairs; petiole and postpetiole dorsolaterally with subdecumbent or suberect stiff or flexous hairs, ventrally usually denudate; ventropostpetiolar process denudate or bearing few scattered, long, flexous hairs, rarely pubescent, metasomal 3 (Abd IV) tergite denudate or bearing either erect or suberect, long or short, flexous pilosity, or combination of both; exception of pubescent metasomal 3 tergite is Andean species from Panama and Venezuela; ventral metasomal 3 denudate, pubescent, or with combination of pubescent and short, scattered, flexous hairs; metasomals 4 to pygidial postergites with few erect or suberect flexous or stiff hairs, often arranged in transverse lines with shorter hairs [species at low elevations]; hypopygium pubescent or not, and often possessing short, subdecumbent, flexous hairs. Brazilian species usually with stiff pilosity shorter than flexous hairs of some Andean and Mesoamerican species, short stiff hairs in Andean *convexus, hirsutus, belti*. Some Andean species may bear flexous or stiff, long or short pilosity; other species may possess abundant flexous pilosity [*batesi*]. Some species almost denudate dorsally and pubescent ventrally, especially on opisthogastral sternites [*eladio*]. Few Brazilian species with some very scarce long flexous hairs on caudal metasoma posttergites.

#### Color

Color usually varies from dark-orange or red-brown appendages, and black meson in some Andean species usually over 600 m of elevation, to completely black in most species found at low elevations. Minor color variations are as follows. Scape and pedicel yellow, orange, red, or brown; eye brown; palps yellow; mandible brown laterodistad; flagellum, tibiae and telotarsi orange-brown; remaining body black.

#### **Male** (Figures 36–45)

Male longer, slender than gyne (Figure 37), ranging from 4.8 mm [COL] to 9.9 mm [*scabriusculus*]. Following traits separate *Procryptocerus* males from others in the Tribe Cephalotini: scape long, subequal to or longer than second funicular segment; postpetiole longer than height; mandibles strongly mesially curvate; head subglobular; posterolateral spines or teeth on dorsopropodeum (Kempf 1951).

#### **Head** (Figures 36–38)

Subglobular, never transverse. Interocular distance shorter than, or subequal to, median head length; mandibles curvate mesially; anteclypeal carina medially weakly notched; clypeus protruded into nasus; frontoclypeal sutures modified in transverse groove between toruli; frontal carina short, divergent caudad; vestigial to obsolete behind eyes; antennal scrobe above eyes; vertex not distinctly deflexed; frons posterior corners distinct to obsolete. Eyes lateral, strikingly protruding, slightly extending dorsad and ventrad, comprising most of head; ocelli protruding in most species dorsally, posteriad to eyes, anteriad to vertexal margin, sometimes assemblaged on ocellar triangle; antenna filiform, 13-segmented; scape subequal to or longer than second funicular segment.

#### **Mesosoma** (Figures 37, 38)

Trunk-shaped, humped; scutum with deeply impressed notauli; anterior branches longer than the posterior medial stem; episternum superior to mesocoxa, usually ecarinate; dorsopropodeum with small, spiniform process posterolaterally; femora moderately concave mesially and incrassate in middle; all segments of legs comparatively long and slender; middle and hind tibiae usually without apical spur.

#### **Metasoma** (Figures 37, 38)

Metasomal 1 (Abd II = petiole) sessile, elongate, subcylindrical. Metasomal 2 (Abd III = postptiole) similar to metasomal 1, somewhat shorter, more incrassate posteriad; metasomal 3 (Abd IV) largest metasomal somite, longer than petiole and post-petiole combined; hypopygium rounded posteriad (Figure 40) [*scabriusculus*], truncate [*batesi*], subtriangular or conic [*adlerzi*]; paramere (Figures 41–43) paddle-shaped, rounded apically, sometimes about same length of caudal metasoma ; volsella (Figure 45) mesiad to paramere, bifurcate into cuspis and digitus volsellaris; cuspis volsellaris mesial to paramere, subcylindrical, sinuate, shorter than digitus, truncate apically; digitus volsellaris mesial to cuspis, compressed, hook-shaped, ventrally bent distad.

#### **Wings** (Figure 46)

Wing shape, venation and cells similar in both male and gyne; male fore wing extending to level of posteriad caudal metasoma; hyaline [*goeldii*], or infumate [*batesi, mayri, impressus, scabriusculus*]. Useful, distinct variations have not been found for discriminating species within *Procryptocerus*. Fore wings with distinct anterodistad stigma; in anteroposterior sequence, proximal (proximad to stigma) longitudinal veins are C (Costa), Sc+R+Rs (Subcosta+Radial+Radiosectorial), M+Cu (Medial+Cubital), and A (Anal). Distad longitudinal veins are R, M and Cu; distad veins do not reach apex of wing; recurrent veins are cu-a (cubital-anal) and m-cu (medial-cubital); cells formed by confluence between longitudinal veins or between longitudinal and recurrent veins; proximally, three cells present: Costal (CC), Basal (BC) or Radial (“Media”), and Subbasal (SBC) or Cubital (“Submedia”); posteriad to stigma, Submarginal-one cell (SMC1) (closed) and Submarginal-two cell (SMC2) (opened) are present; Discal cell-one (DC1) posteriad to Submarginal-one and distad to Basal cell (or Radial cell), formed by confluence of M, Cu, Rs and m-cu. Distal field without cells. Hind wing possessing proximally two distinct longitudinal veins: R+Rs and M+Cu; cu-a is basad in proximal field; Basal cell BC (or Radial cell) closed distally by M vein; Subbasal SBC cell (or Cubital cell) closed distally by cu-a and posteriorly by 1A; distal field without distinct veins.

Along with different shapes of discoidal and first submarginal cells on anterior wings, main characters that separate species are variations within external and internal genitalia, which contain well-developed hypopygium, volsellae, and parameres (Kempf 1951).

## Discussion

### **Torulus vs. Annulus (antennalis)** (Figures 4, 14)

Gauld and Bolton (1988) consider the torulus to be the socket, or the cephalic foramen, in which the antennal condylar bulb inserts. Bolton (1994) considers torulus to be the small annular sclerite that surrounds the antennal socket. Torre-Bueno (1989) considers the annulus (antennalis) to be the ring sclerite of the head into which the basal segment of the antenna is inserted. Gordh and Headrick (2002) consider annulus to be the antennal sclerite forming a sclerotized ring on the head into which the basal segment (scape) of the antenna is inserted. We follow Bolton (1994).

### **Mesosoma** (3, 9)

For the second tagma, the term “alitrunk” (ali = wings) has been proposed to avoid confusion with “thorax” (Gauld and Bolton 1988, Bolton 1994, Wilson 2003). Nevertheless, a similar confusion could occur between metasoma and “abdomen”. “Alitrunk” is not recommended since worker ants do not possess wings, and mesosoma is well characterized in Apocrita (see Nauman 1991).

### Notopropodeal fusion

The mesosoma comprises the thorax plus the propodeum, the tergite of Abd I fused to the thorax. The pleurites and the sternite of Abd I are entirely reduced and the tergite remains. In workers of ants, the notai and propodeal sclerites are usually fused forming a tergal (notai) fusion between the notum and propodeum. This condition is a notopropodeal fusion. Externally, it is usually impossible to recognize the structures involved in the fusion. The line of fusion may be indistinct (notopropodeal fusion usually convex), obsolete or differently marked by a suture, groove, impression, depression, etc. The line of fusion has different names in the literature, such as “propodeal suture” (a suture on the propodeum), “metanotal suture” (a suture on the metanotum), “metanotal groove” (a groove on the metanotum), “metanotal impression” (an impression on the metanotum), “metapropodeal suture” (a suture on the posterior [meta] region of propodeum), antepropodeal suture, metanotal area, etc. Since these terms make reference to the line of fusion, we recommend using the adjective *notopropodeal* in reference to the line of fusion; for instance: notopropodeal *suture*, notopropodeal *groove* (Figure 3), notopropodeal *convexity*, notopropodeal *impression*, notopropodeal *excavation*, or otherwise make reference to the notopropodeal fusion to describe specific characters, such as *notopropodeal fusion flat, notopropodeal fusion convex*, etc. In several groups, e.g. some *Camponotus*, the metanotum is clear and so are the mesometanotal suture and the metanotal-propodeal suture. In those cases, a notopropodeal fusion is not apparent. Occasionally, a mesometanotal fusion or a metanotal-propodeal fusion can also be identified in a very few cases.

### **Sculpture** (Figures 14–34)

*Procryptocerus* species bear both micro- and macrosculpture. Microsculpture (micropunctulate, micropunctate, microimbricate, microstrigate, “dotted”) covers the background of the cuticle. Microsculpture may be present on the elevations or impression of the macrosculpture, usually when the macrosculpture (e.g. costae, striae) is glossy (shiny), or, more commonly, in smooth surfaces devoid of macrosculpturing. Micropunctulae and microimbricae are fairly different, but require close inspection to interpret. The micropunctulae condition occurs as microscopic pricks more common on opaque surfaces, whereas microimbricae are either overlapping microscales (as tiles on a roof) or microreticulae (“dotted”) that give an overlapped appearance. The latter are more common on glossy surfaces. Macrosculpture can be divided into impressed, superficial, raised and combined sculpturing with the following states. 1. Impressed macro sculpture: circular or oblong (punctate, foveate, foveolate); linear (furrow-like) (furrowed, grooved, sulcate). 2. Superficial macro sculpture (non-impressed cuticle, surface spaces between costae or carinae): linear spaces delimited by costae or ridges costae or ridges are low and same width as striae- (striate, striolate, strigate, strigulate); non-linear spaces delimited by costae or ridges (reticulate, areolate). 3. Raised macro sculpture (carinate, carinulate, costate). 4. Combinations between impressed and raised macrosculpture: canalicular (porcate, scrobiculate); polygonal and irregularly polygonal (alveolate), or irregular porcae, irregular reticulae, irregular rugocostae-alveolate (clathrate).

Striate sculpturing refers to longitudinal lines on a non-impressed cuticle, running parallel between thin and low elevated costae or costulae; the costae (or costulae) and lines are narrow and about the same width. The sculpture should be named striate-costulate, but it is customary to call it striate. Striate is one of the most common forms of sculpturing in *Procryptocerus*; it is often present on the metasomal 3 (first opisthogastral) tergite. Costate sculpturing refers to costae (elevated ridges rounded at their edges, dim. costulae) in general running parallel or quasi parallel to each other, the interspaces are wider than costae. Rimose refers to *nearly* parallel excavations (rimae), often narrow, short or long, in the shape of wavy cracks, running into each other (Gordh and Headrick 2000); elevations between rimae are vermiculate, often wide and flat at their ridges, which are usually micropunctulate. Rimae refer to the longitudinal fissures, crack-shaped interspaces; ridges refer to the elongated elevations (costae). The costae are wavy. The combination between rimae and flat ridges, running in anastomosis, produces rimose (dim. rimulose) or rivose (dim. rivulose) sculpturing. Combinations of character states, shuch as rimose-vermiculate, or rivose-vermilaculate could be more specific.

Punctures are slightly impressed points (pricks) on the cuticle that appear to be made by a needle (Gordh and Headrick 2000). Punctures constitute the smallest circular-macrosculpture. Derived adjectives describing this sculpture are punctate (with punctures), puncticulate (sparcely punctate), punctulate (closely punctate). When puncticulate and punctulate are present on the same surface, the difference between the sculptures is clear. When only one is present, the terms are interchangeable. When densely punctate, the cuticle has a farinose texture. Dots (dotted sculpturing) are non-impressed circular marks, they are superficial, rounded, and the same size as micropunctures. A costate integument emphasizes the costae and not the interspaces (striae or sulci); in these cases spaces between costae are in general wider than the costa edges and not impressed. The sculpture is porcate when a set of combinations of costae and impressions between costae are present, forming canalicular (sulcal) spaces. Anastomosed porcae are porcae that run into each other. Scrobiculate are surfaces where scrobiculae (parallel, short porcae) are uniformly organized in a contiguous, chainlike series. When there are septae within striae, the sculpturing is reticulate (quadri- or quasi quadriculate) [*belti* frons] or areolate (polygonal or quasi polygonal) [*scabriusculus* frons posteriad]. In a subsecuent stage there are septae within a porcate surface, and the sculpturing is alveolate or clathrate. Alveolae are lacunose, impressed spaces delimited by irregular rugae or “costae” with sharp rims at their edges. The alveolae are regular or irregular polygons, and the sculpturing is called alveolate. Alveolate cuticle is often present on the posterior frons and petiole. Surfaces having alveolate sculpturing in *Procryptocerus* form landscapes of lacunae between ridges (ranges of “hills”) containing sharp or obtuse edges. Clathrate sculpturing refers to the most complex, irregular combination of irregular porcae and transversal septae (crossing costae), forming deep, alveolate, cancelled holes of different and irregular diameter. In clathrate sculpture, the costae run anteroposteriorly in irregular fashion prevailing over the short transverse costae forming the septae. High density of alveolae conform clathrate sculpture. This characteristic is the most important one to differentiate clathrate and reticulate sculpturing, which could be apparently similar when both are present on frons and when looking at them through a common microscope. Clathrate sculpture may be present on the frons and mesonotum [*mayri, batesi, clathratus*]. Reticulate sculpture is usually present on frons only [*belti, convexus, hirsutus*].

When a combination of sculptures is present, it is useful to hyphenate two, sometimes three, different words qualifying sculpture (e.g. rugo-costate, costate-foveate, foveate-reticulate, micro-striolate-imbricate). In general, when differences between proportions occur, the first word should emphasize the most common sculpture, or emphasize the first sculpture when referring to an anteroposterior (or any directionality) sequence of the sculpture present on any surface. Discriminating thickness of raised sculpture (e.g. costate, carinate) and width of circular, impressed sculpture (e.g. punctate, foveate, foveolate) is often not clear when only one of these types of sculpture is present. In those cases, the closest terms might be used interchangeably (e.g. foveate or foveolate, costate or costulate, rimose or rimulose). Discrimination of those sculptures is easier when several types of sculpture are present in the same area of an ant.

Confusion occurs between sculpture texture (appearance) and sculpture structure (constitution). To recognize the constitution (nature) of the sculpture, textures (e.g. leathery, farinose, rugous, coriarious, corticinus, etc.) should be avoided. Appearance strongly depends upon the “momentary” criterion of the researcher or interpreter, and magnification, light or system (microscope, SEM images, photomontage) used to recognize it. Nonetheless, when using a common microscope or photomontage images, the appearance is sometimes quite distinct with some descriptive forms (e.g. politus, shiny, glossy, farinose). It is best to use SEM images of sculpture (For instance see http://www.evergreen.edu/ants/genera/AntsofCostaRica.html). On the other hand, drawings are the best way to convey information about boundaries of sclerites.

Sculpturing within *Procryptocerus* is a rich source of characters, which is helpful in stablizing and recognizing the identity of species and could permit the formalization of hypotheses of evolutionary trends.

**Figures 17–21.**
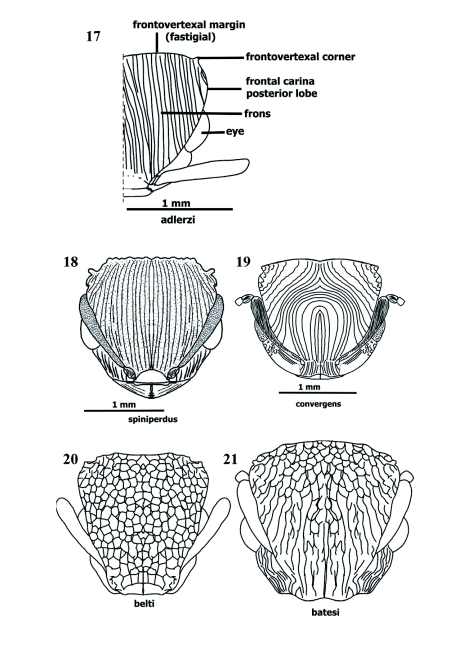


**Figures 17–25.**
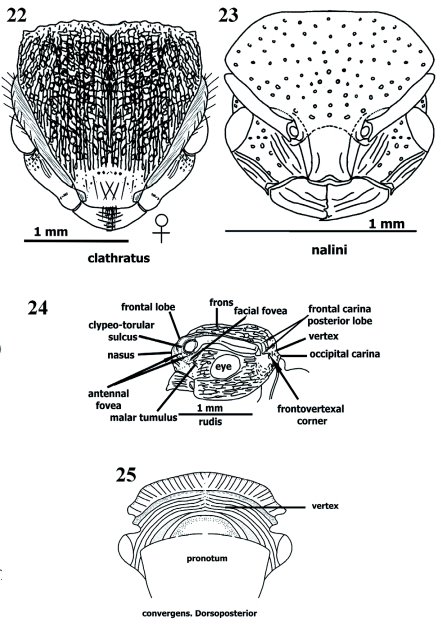
Sculpture on head. 17–23 frontal (dorsoanterior); 24 lateral (profile); 25 dorsoposterior. 17 costate, 18 costate, concentricus in middle, 19 diverging costate posteriad, concentricus anteriad, 20 reticulate, 21 clathrate posteriad and in middle, rugocostate anterolaterad, anastomosate anteromesiad, 22 clathrate, 23 foveate or foveolate, 24 parietal costate or rugocostate, frons clathrate, 25 vertex strigate. High quality figures are available online.

**Figures 26–34.**
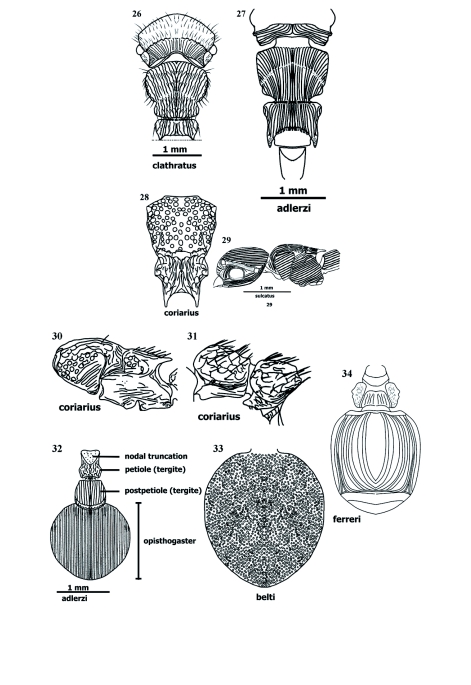
Sculpture on pro, meso and metasoma. 26 vertex striate (or longitudinally costate), mesosoma costate-vermiculate (or rimosus), 27 vertex strigate (or transversally costate), mesosoma costate, 28 (based on Longino 2006): promesonotum foveate, dorsopropodeum costate-porcate, 29 costate-sulcate, 30 (based on Longino 2006): lateropropropodeum supra foveate, infra costate, pleuron rugocostate, 31 (based on Longino 2006): rugocostate-alveolate, 32 petiole rugocostate, cinctus 2 scrobiculate, postpetiole costate (or porcate when interspaces are deep), metasomal 3 (Abd IV) striate-puncticulate (densely punctate), 33 metasomal 3 (Abd IV) tergite punctate, 34 metasomal 3 (Abd IV) sternite costulate-concentricus, glossy in middle. High quality figures are available online.

**Figure 35.**
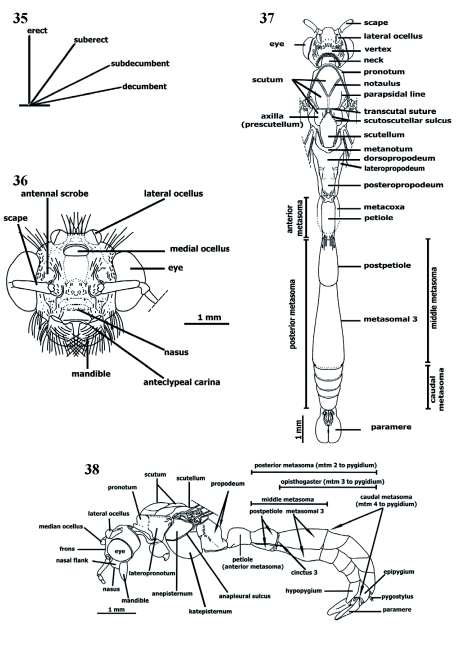
General orientation of hairs. **Figure 36.** *Procryptocerus scabriusculus*. Male. Head. Frontal (anterodorsal) view. **Figure 37.** *Procryptocerus scabriusculus*. Male. Dorsal view. **Figure 38.** *Procryptocerus scabriusculus*. Male. Lateral view; mtm: metasomal. High quality figures are available online.

**Figures 39–45.**
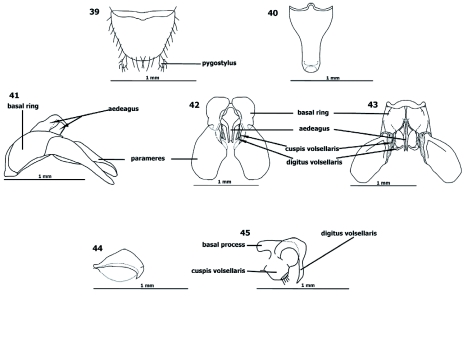
*Procryptocerus scabriusculus*. Terminalia. 39: epipygium; 40: hypopygium; 41, 42, 43: genitalia, 41 : lateral, 42: dorsal, 43: ventral; 44: aedeagus; 45: volsella. High quality figures are available online.

**Figure 46.**
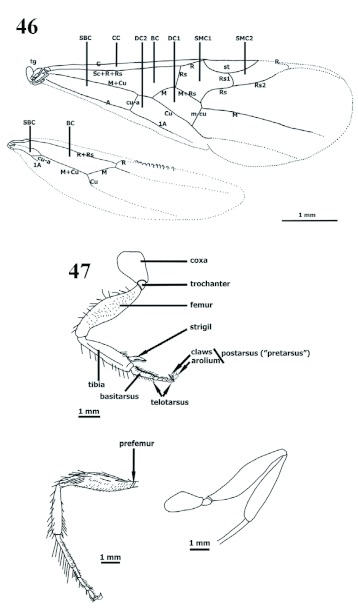
Male wings. Veins: C (Costa), Sc+R+Rs (Subcosta+Radial+Radiosectorial), M+Cu (Medial+Cubital), A (Anal), cua (cubital-anal), m-cu (medial-cubital). Cells: CC (Costal Cell), BC (Basal Cell) (or Radial), SBC (Subbasal Cell) (or Cubital), SMC1 (Submarginal Cell 1), SMC2 (Submarginal Cell 2), DC1 (Discal Cell 1), DC2 (Discal Cell 2); st: stigma. **Figure 47.** Male legs. Above: foreleg; lower left: middle leg; lower right: hind leg (vestiture not drawn). High quality figures are available online.

## Abbreviations

see Glossary (available online)

## References

[bibr01] Agosti D, Majer JD, Alonso LE, Schultz T (2000). *Ants. Standard Methods for Measuring and Monitoring Biodiversity*..

[bibr02] Bohart RM, Menke AS (1976). *Sphecids Wasps of the World*..

[bibr03] Bohart RM, Stange LA (1965). *A Revision of the Genus Zethus Fabricius in the Western Hemisphere (Hymenoptera: Eumenidae)*..

[bibr04] Bolton B (1990). Abdominal characters and status of the Cerapachyine ants (Hymenopera, Formicidae).. *Journal of Natural History*.

[bibr05] Bolton B (1994). *Identification Guide to the Ant Genera of the World*..

[bibr06] Bolton B (2003). Synopsis and Classification of Formicidae.. *Memoirs of the American Entomological Institute*..

[bibr07] Bolton B, Snelling RR, Fisher BL, Ward PS (2007). How to conduct large-scale taxonomic revisions in Formicidae. *Advances In Ant Systematics (Hymenoptera: Formicidae): Homage To E. O. Wilson — 50 Years Of Contributions*..

[bibr08] Bolton B, Alpert G, Ward PS, Nastkrecki P (2006). Bolton's Catalogue of Ants of the World: 1758–2005..

[bibr09] Brown RW (1979). *Composition of Scientific Words*..

[bibr10] Baroni Urbani C, de Andrade ML (2003). The ant genus *Proceratium* in the extant and fossil record (Hymenoptera: Formicidae).. *Museo Regionale di Sciénze Naturali Monografie*.

[bibr11] de Andrade ML, Baroni Urbani C (1999). Diversity and Adaptation in the Ant Genus *Cephalotes*, Past and Present.. *Stuttgarter Beitrage zur Naturkunde*. Serie B (Geologie und Palaontologie), Nr. 271..

[bibr12] Emery C (1887). Catalogo delle formiche esistenti nelle collezioni del Museo Civico di Genova. Parte terza. Formiche della regione Indo-Malese e delláustralia.. *Annali del Museo Civico di Storia Naturale di Giacomo Doria*.

[bibr13] Wytsman P, Emery (1924). (1922). Hymenoptera. Fam. Formicidae. Subfam. Myrmicinae.. *Genera Insectorum*, fasc..

[bibr14] Gauld ID, Bolton B (1988). *The Hymenoptera*..

[bibr15] Gordh G, Headrick DH (2001). *A Dictionary of Entomology*..

[bibr16] Harris KA (1979). *A Glossary of Surface Sculpturing*..

[bibr17] Hölldobler B, Wilson EO (1990). *The ants*..

[bibr18] Kempf WW (1951). A taxonomic study on the ant tribe Cephalotini (Hymenoptera: Formicidae).. *Revista de Entomologia*.

[bibr19] Kempf WW (1957). Sôbre algumas espécies de *Procryptocerus* com a descrição duma espécie nova (Hymenoptera, Formicidae).. *Revista Brasileira de Biologia*.

[bibr20] Kukalová-Peck J, Naumann ID, Carne PB, Lawrence JF, Nielsen ES, Spradbery JP, Taylor RW, Whitten MJ, Littlejohn MJ (1991). Fossil History and the Evolution of Hexapod Structures. (Chapter 6). *The Insects of Australia*..

[bibr21] Longino J.T. (2006). http://academic.evergreen.edu/projects/ants/AntsofCostaRica.html..

[bibr22] Longino JT, Snelling RR (2002). A Taxonomic revision of the *Procryptocerus* (Hymenoptera: Formicidae) of Central America. Contributions in Science, Number 495..

[bibr23] Mackay WP, Vinson SB (1989). A Guide to species identification of New World Ants (Hymenoptera: Formicidae).. *Sociobiology*.

[bibr24] Mackay WP, Mackay EE (2003). *The ants of New Mexico (Hymenoptera: Formicidae)*..

[bibr25] Mackay WP, Mackay EE (2006). A new species of the ant genus *Pachycondyla* F. Smith, 1858 from Ecuador (Hymenoptera: Formicidae).. *Myrmecologische Nachrichten*.

[bibr26] Naumann ID, Naumann ID, Carne PB, Lawrence JF, Nielsen ES, Spradbery JP, Taylor RW, Whitten MJ, Littlejohn MJ (1991). Hymenoptera (Wasps, bees, ants, sawflies).. *The Insects of Australia*..

[bibr27] Torre-Bueno JR de la (1989). The Torre-Bueno Glossary of Entomology, compiled by SW Nichols, New York Entomological Society..

[bibr28] Perrault GH (2004). Étude morphoanatomique et biométrique du métasoma antérieur des ouvrières. Contribution à la systématique et à la phylogénie des fourmis (Hymenoptera : Formicidae).. *Annales Société entomologique de France*.

[bibr29] Schultz TR, Alonso LE, Agosti D, Majer JD, Alonso LL, Schultz TR (2000). Glossary.. Ants. Standard Methods for Measuring and Monitoring Biodiversity..

[bibr30] Snodgrass RE (1993). *Principles of Insect Morphology*..

[bibr31] Sparks SD (1941). Surface anatomy of ants.. *Annals of the Entomological Society of America*.

[bibr32] Triplehorn CA, Johnson NF (2005). Borror and Delong's Introduction to the study of insects 7^th^ edition..

[bibr33] Ward PS (1999). Systematics, biogeography and host plant associations of the *Pseudomyrmex viduus* group (Hymenoptera: Formicidae), *Triplaris*- and *Tachigalia*-inhabiting ants.. *Zoological Journal of the Linnean Society*.

[bibr34] Ward PS (2004). Ant Morphology. The Ant Course No.4. August 2004, Costa Rica..

[bibr35] Ward PS, Downie DA (2005). The ant subfamily Pseudomyrmecinae (Hymenoptera: Formicidae): phylogeny and evolution of big-eyed arboreal ants.. *Systematic Entomology*.

[bibr36] Ward PS, Zhang ZQ, Shear WA (2007). Phylogeny, classification, and species-level taxonomy of ants (Hymenoptera: Formicidae).. Linnaeus tercentenary: Progress in invertebrate taxonomy. Zootaxa 1668..

[bibr37] Wheeler DE (1984). Behavior of the ant, *Procryptocerus scabriusculus* (Hymenoptera: Formicidae), with comparisons to other cephalotines.. *Psyche*.

[bibr38] Wheeler QD (2008). Undisciplined thinking: morphology and Henning's unfinished revolution.. *Systematic Entomology*.

[bibr39] Wilson EO (2003). *Pheidole* in the New World: A Dominant, Hyperdiverse Ant Genus..

